# Coronary pathophysiology in idiopathic pulmonary arterial hypertension

**DOI:** 10.1172/jci.insight.194613

**Published:** 2026-01-22

**Authors:** Erin Boland, Michael G. Freeman, David S. Corcoran, Thomas J. Ford, Barry Hennigan, Damien Collison, Aida Llucià-Valldeperas, Frances S. de Man, Kanarath P. Balachandran, Martin Johnson, Colin Church, Colin Berry

**Affiliations:** 1 British Heart Foundation Glasgow Cardiovascular Research Centre, University of Glasgow, Scotland, United Kingdom.; 2 West of Scotland Heart and Lung Centre, Golden Jubilee National Hospital, Glasgow, Scotland, United Kingdom.; 3 Central Heart, Toukley, New South Wales, Australia.; 4 Department of Pulmonary Medicine, Amsterdam University Medical Centre, Vrije Universiteit Amsterdam, PHEniX Laboratory, Amsterdam, Netherlands.; 5 Amsterdam Cardiovascular Sciences, Pulmonary Hypertension and Thrombosis, Amsterdam, Netherlands.; 6 Department of Cardiology, Royal Blackburn Teaching Hospital, East Lancashire Hospitals NHS Trust, Lancashire, United Kingdom.; 7 Scottish Pulmonary Vascular Unit and Cardiology Department, Golden Jubilee National Hospital, Glasgow, Scotland, United Kingdom.

**Keywords:** Cardiology, Pulmonology, Vascular biology, Cardiovascular disease, Hypertension, Microcirculation

## Abstract

**BACKGROUND:**

Idiopathic pulmonary arterial hypertension (IPAH) alters right ventricular size and function, curtailing life expectancy. Patients may experience angina and myocardial ischemia. However, the underlying mechanisms are poorly understood.

**METHODS:**

This study had a cross-sectional, case-control design. Patients with IPAH undergoing right heart catheterization were prospectively enrolled and underwent functional testing during coronary angiography using a dual pressure/temperature-sensitive guidewire. Cardiovascular MRI measured left and right ventricular mass and function. Right ventricular tissue from individuals with end-stage PAH and control individuals were analyzed for pathophysiology.

**RESULTS:**

Eleven IPAH and 15 control participants completed the protocol: 73% of IPAH patients had an elevated index of microcirculatory resistance (IMR > 25) and 55% had reduced coronary flow reserve (CFR < 2.0). Mean IMR was significantly higher in IPAH participants (39.2 ± 27.0 vs. 15.3 ± 5.0, *P* = 0.002), whereas mean CFR was lower (2.8 ± 2.1 vs. 4.0 ± 1.4; *P* = 0.077). Paired right coronary artery/ventricular measurements (*n* = 6) revealed IMR positively correlated with right ventricular mass (*r* = 0.91, *P* = 0.12) and negatively with CFR (*r* = –0.82, *P* = 0.046). Compared with controls (*n* = 5), PAH participants (*n* = 4) had reduced right ventricular capillary density, increased cardiomyocyte area, and increased mural area in pre-capillary arterioles.

**CONCLUSION:**

Invasive coronary function testing was feasible and safe in IPAH. Coronary microvascular dysfunction was prevalent in IPAH and correlated with increased right ventricular mass. Histopathology revealed vascular rarefaction and remodeling of pre-capillary arterioles.

**FUNDING:**

The British Heart Foundation (BHF) (PG/18/6134217) and the Golden Jubilee Research Foundation.

## Abstract 2

Dysfunction of the mirovasculature in the heart is a common finding in patients with idiopathic pulomary arterial hypertension.

## Graphical abstract

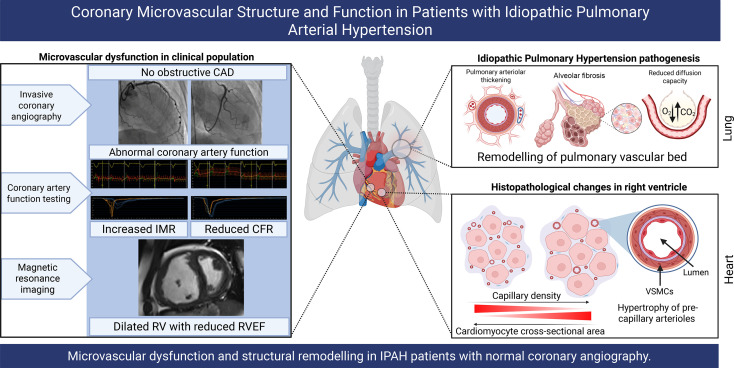

## Introduction

Idiopathic pulmonary arterial hypertension (IPAH) is a rare condition involving progressive pulmonary microvascular obstruction (1, 2). IPAH may have a genetic basis, although the etiology remains unclear for many patients. The disease has a female preponderance, with a mean age of 50 years at diagnosis (1, 2) and a poor prognosis. The onset of right heart failure heralds a terminal phase with survival limited to approximately 6 months; sudden cardiac death often occurs in the advanced stages (3, 4). This condition is classified in practice guidelines as group 1 PAH (3, 4).

Angina symptoms and myocardial ischemia are recognized complications of IPAH; however, the underlying mechanisms are not understood. Pulmonary hypertension increases right ventricular afterload, resulting in right ventricle hypertrophy. Coronary perfusion pressure should increase to maintain myocardial perfusion (5). Systolic right coronary artery flow may be impaired in IPAH due to extravascular compression from the hypertrophied right ventricle, potentially resulting in myocardial ischemia (5). In addition, angina may potentially occur due to coexisting coronary atherosclerosis and/or microvascular dysfunction (6). The pathophysiology of microvascular dysfunction may involve anatomical abnormalities (e.g., medial wall thickening, capillary rarefaction) or functional abnormalities of the coronary microvasculature (e.g., impaired vasodilation or abnormal vasoconstriction) or both, leading to a supply-demand mismatch in myocardial blood flow at rest or during physiological stress (6). Right ventricular ischemia is associated with adverse remodeling and dysfunction in patients with IPAH (7).

The pathogenesis of IPAH leading to right ventricular hypertrophy indicates microvascular dysfunction may develop (8, 9). Occlusive coronary microvascular disease and reduced angiogenesis have been observed in animal models of pulmonary hypertension; however, to our knowledge, coronary microvascular function has not been described (10).

The study aims were, first, to invasively assess coronary microvascular function in patients with confirmed IPAH; second, to assess the safety of this approach in this population; third, to characterize the microvascular histopathology in right ventricle samples obtained from patients with end-stage IPAH and controls.

We hypothesized that abnormalities in coronary microvascular structure and function in patients with IPAH are more prevalent than controls, and that parameters of microvascular function correlate with measures of IPAH disease severity.

## Results

### Study population

The participants’ characteristics for both the IPAH and noncardiac symptom control group are outlined in Table 1. In the IPAH group of 11 individuals, the age was 47 ± 15 years (mean ± SD), 10 (91%) of the individuals were White, and 1 (9%) individual was Asian. Eight (73%) individuals were female, and 3 (27%) were male. The mean pulmonary arterial pressure was 51 ± 8 mmHg. The control group included 15 individuals, of which 11 (73%) were females (age 58.3 ± 9.1 years), who prospectively underwent invasive endotyping during the study period and received a final diagnosis of noncardiac chest pain (Table 1). There is a greater incidence of IPAH within the female population, which is reflected in the percentages of male and female participants included in this study. Sex stratification analyses were not performed due to the statistical power requirement of our study group size.

### Screening

**Figure 1:**
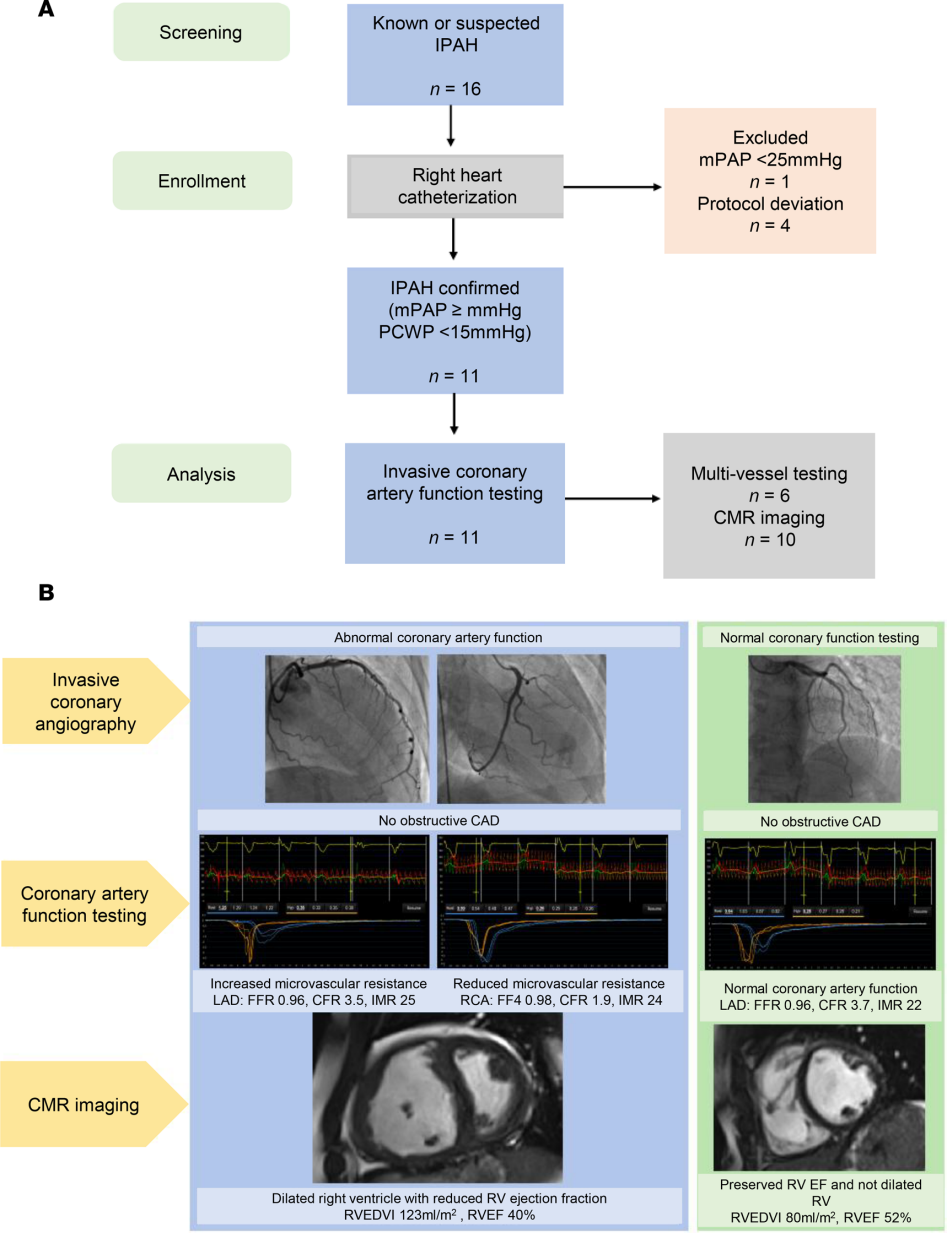
Flow diagram and schematic of invasive and noninvasive phenotyping. (**A**) Participant enrollment and exclusions. Patients with IPAH undergoing right heart catheterization were assessed for eligibility. Numbers completing invasive coronary function testing, multi-vessel assessment, and cardiovascular MRI are shown. Representative examples of invasive and noninvasive investigations. Left panels show findings from a patient with IPAH demonstrating abnormal coronary physiology despite no obstructive CAD on invasive coronary angiography. Coronary function testing shows increased microvascular resistance in the LAD and RCA, consistent with coronary microvascular dysfunction (e.g., elevated IMR, reduced CFR). Corresponding CMR imaging of a dilated right ventricle with reduced ejection fraction. In contrast, right panels show control data with normal coronary angiography, preserved coronary physiological indices, and normal right ventricular size and systolic function on CMR. mPAP, mean pulmonary artery pressure; PCWP, pulmonary capillary wedge pressure; CMR, cardiac magnetic resonance; LAD, left anterior descending artery; RCA, right coronary artery; IMR, index of microcirculatory resistance corrected for central venous pressure; CFR, coronary flow reserve; FFR, fractional flow reserve; RVEDVi, right ventricular end-diastolic volume indexed to body surface area; RVEF, right ventricular ejection fraction.

Sixteen patients were recruited from January 2016 to September 2018 (Figure 1A). After right heart catheterization, 1 patient was excluded because the diagnostic criteria for IPAH were not met. Four (27%) participants were subsequently excluded due to protocol deviations (failed to complete the invasive coronary angiogram: *n* = 2 withdrew consent prior to the right heart catheterization, *n* = 1 unable to gain radial artery access, *n* = 1 equipment technical error), and 11 were included in the final analysis (Figure 1A). Ten participants had baseline cardiovascular MRI data available (*n* = 1 incomplete scan due to claustrophobia).

### Invasive coronary function testing

All participants had at least 1 coronary artery assessed: left anterior descending artery (*n* = 8); right coronary artery (*n* = 10); left circumflex (*n* = 5) (Table 2); multivessel measurements (*n* = 6); or all 3 arteries measured (*n* = 5) (Supplemental Table 2). All participants had dominant right coronary arteries. Eight (73%) and 6 (55%) participants with IPAH had an abnormal index of microcirculatory resistance (IMR ≥ 25) or coronary flow reserve (<2.0), respectively, in any artery (Table 2). The mean ± SD (range) of the IMR and coronary flow reserve were 36 ± 26 AU (14–104) and 2.8 ± 2.1 AU (1–10), respectively.

In the control individuals, all coronary function tests were performed in the left anterior descending artery. The mean ± SD (range) IMR and coronary flow reserve were 15.3 ± 5.0 AU (5.3–23.0) and 3.9 ± 1.4 AU (2.1–6.6), respectively (Table 3).

In comparison between groups, in the participants with IPAH, the IMR was significantly increased compared with the control participants (mean difference = 23.9 [95% CI, 9.6–38.2], *P* = 0.002) and coronary flow reserve was reduced, but this difference was not statistically significant (mean difference: –1.2 [range, –2.4 to –0.1], *P* = 0.08). Fractional flow reserve was higher in the participants with IPAH (mean difference 0.07 [range, 0.04–0.10], *P* < 0.001) (Table 3), consistent with the angiographic findings of minimal coronary atherosclerosis.

### Multivessel coronary physiology and cardiovascular MRI

Coronary microvascular function was similar when assessed in the left anterior descending and right coronary arteries (*n* = 6 participants with paired measurements; Supplemental Table 2).

### Cardiovascular MRI and coronary artery function

Compared with the control population, the participants with IPAH had lower left and right ventricular ejection fractions, higher right ventricle volume and mass, and higher myocardial native T1 relaxation times (ms) (Table 4). A strong positive correlation was found between the IMR_cor_ measured in the right coronary artery and right ventricle mass (*r* = 0.91, *P* = 0.12), and a negative correlation was found between right coronary artery flow reserve and right ventricle mass (*r* = –0.82, *P* = 0.046) (Supplemental Figure 1). Clinically relevant changes in left ventricular functional parameters were also affected within the IPAH group compared with non-IPAH controls. The left ventricular end-diastolic pressure in the IPAH group was 11.5 ± 5.0 mmHg versus 7.1 ± 3.1 mmHg in non-IPAH controls (*P* = 0.021). The left ventricle ejection fraction was 55.8% ± 5.2% in the IPAH group versus 65.9% ± 7.1% in non-PAH controls (*P* = 0.001).

### Safety

There were no adverse events related to the invasive coronary function testing when performed as an adjunctive procedure to right heart catheterization. Intracoronary adenosine was well tolerated.

### Follow-up

Over a follow-up period of at least 9 years, 6 (55%) participants with IPAH died. The mean age at death was 58.3 ± 19.1 years, occurring 5.3 ± 3.4 years after study recruitment.

### Right ventricular histology in PAH and controls

#### Participant characteristics.

To investigate whether a structural coronary endotype linked to microvascular dysfunction was evident in patients with PAH, right ventricle tissues from deceased or transplanted patients with PAH (*n* = 4) were analyzed and compared with right ventricle tissue from deceased healthy individuals (*n* = 5). The characteristics of these individuals are summarized in Supplemental Table 1. The average age of patients in the control group was 42.4 ± 16.3, and the PAH group had an average age of 62.3 ± 7.5. The control individuals had no comorbidities, whereas the PAH group exhibited additional cardiovascular risk factors (Supplemental Table 1). None of the individuals in either group had a documented history of ischemic heart disease or angina. All patients with PAH were classified as New York Heart Association (NYHA) class IV (*n* = 4) and 2 of these individuals had documented N-terminal pro b-type natriuretic peptide (NT-proBNP, 13,970 ± 5,970 ng/L). Cardiovascular phenotyping was unavailable for the deceased controls.

#### Immunohistofluorescence of right ventricular tissue.

**Figure 2:**
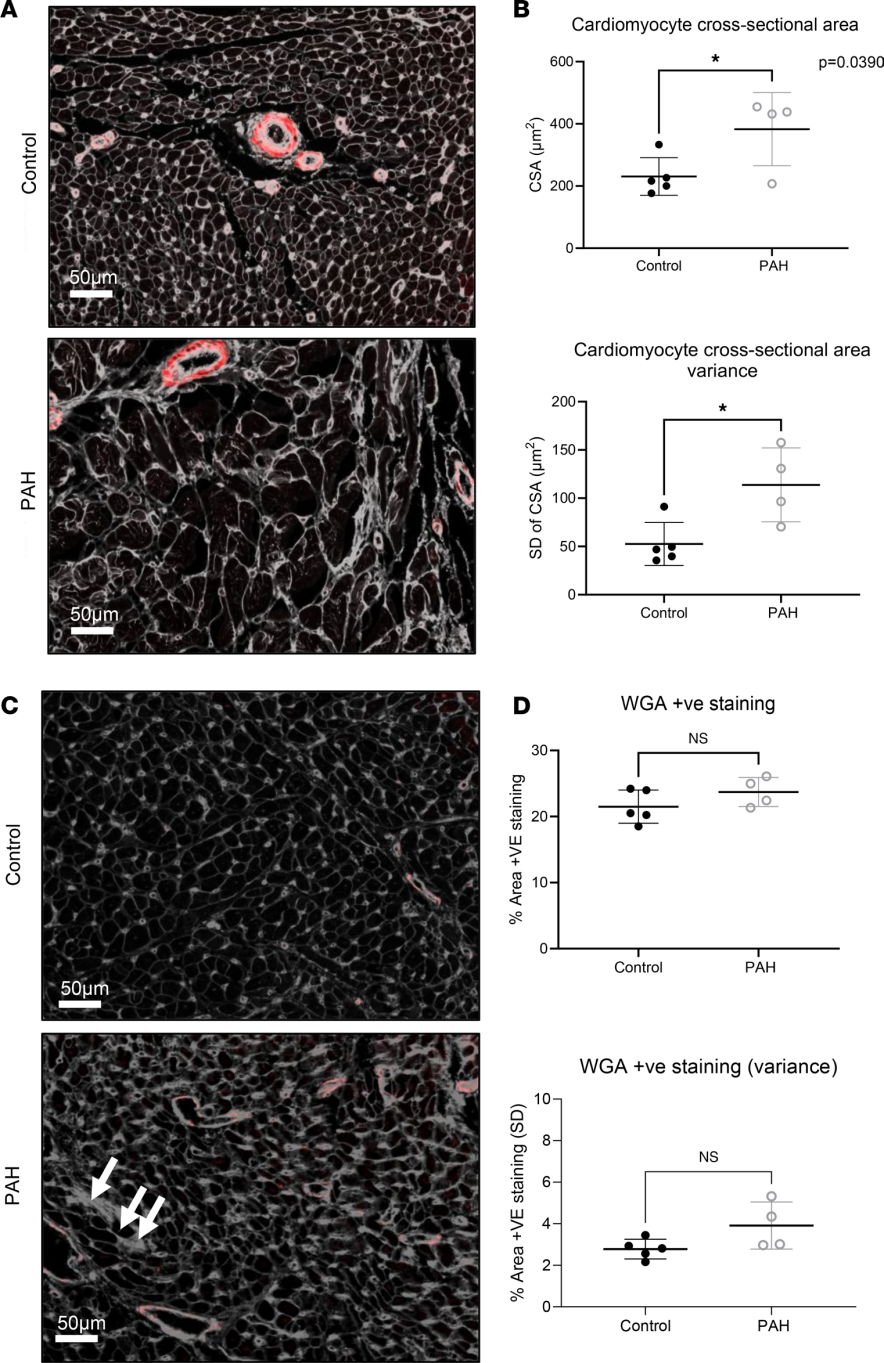
Histological analysis of cardiomyocyte cross-sectional area. (**A**) Representative images of right ventricular tissue stained with WGA (gray) and αSMA (red) from the PAH and control patient groups. (**B**) Quantification of cardiomyocyte cross-sectional area (CSA) revealed a significant difference in cardiomyocyte size between groups (control CSA = 230.7 μm^2^, PAH CSA = 382.9 μm^2^, unpaired 2-tailed *t* test, *P* = 0.0390, control *n* = 5, PAH *n* = 4). Further analysis of the variability in cardiomyocyte size revealed significantly greater variance within the PAH group indicative of increased heterogeneity in cardiomyocyte size groups (control SD = 52.63 μm^2^, PAH SD = 113.8 μm^2^, unpaired 2-tailed *t* test *P* = 0.0192, control *n* = 5, PAH *n* = 4). (**C**) WGA staining revealed regions of interstitial fibrosis within the right ventricle of PAH hearts and surrounding the vasculature (white arrows). (**D**) Quantification of WGA-positive area stained in control and PAH right ventricles revealed a significant difference between the 2 patient groups (control SD = 52.63 μm^2^, PAH SD = 113.8 μm^2^, unpaired 2-tailed *t* test *P* = 0.0192, control *n* = 5, PAH *n* = 4). *P < 0.05. Scale bars: 50 μm.

**Figure 3:**
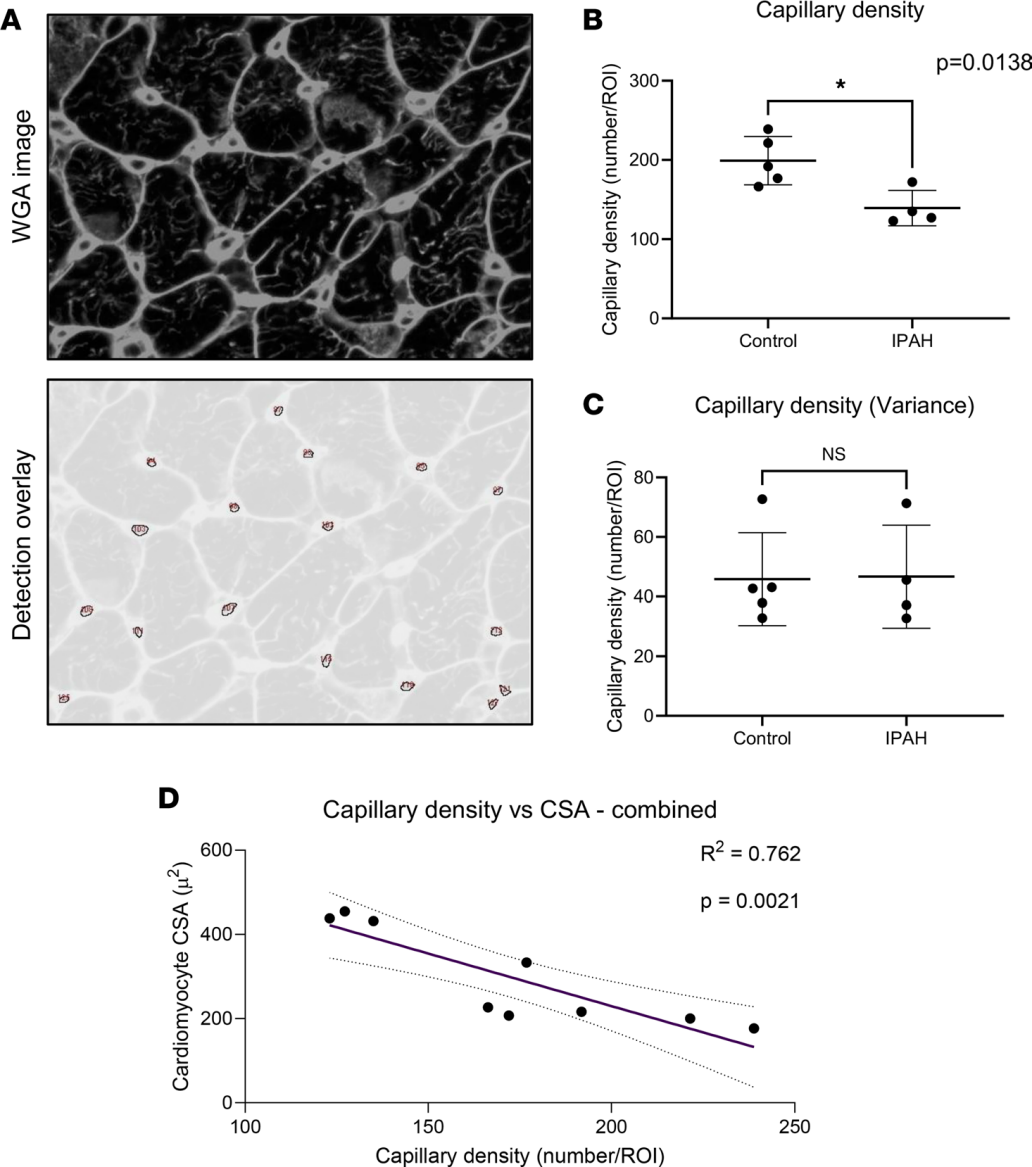
Patients with IPAH display reduced capillary density with a strong correlation to cardiomyocyte hypertrophy. (**A**) Overlay of capillary density analysis quantified via the ImageJ Analyze Particles plug-in identifying capillary structures. (**B**) Quantification of capillary density revealed significantly decreased right ventricular capillary density in patients with IPAH compared with controls (controls 199/ROI, IPAH 139.3/ROI, unpaired 2-tailed *t* test, *P* = 0.0138). (**C**) Quantification of variance between IPAH and control groups showed no difference in capillary density variability (controls 45.86/ROI, IPAH 46.69/ROI, unpaired 2-tailed *t* test, *P* = 0.9414). (**D**) Correlation between capillary density and cardiomyocyte cross-sectional area (CSA) between all patients identified correlation between capillary density and CSA across both groups (*R*^2^ = 0.762, *P* = 0.0021); 95% CI also displayed (dashed lines).

**Figure 4:**
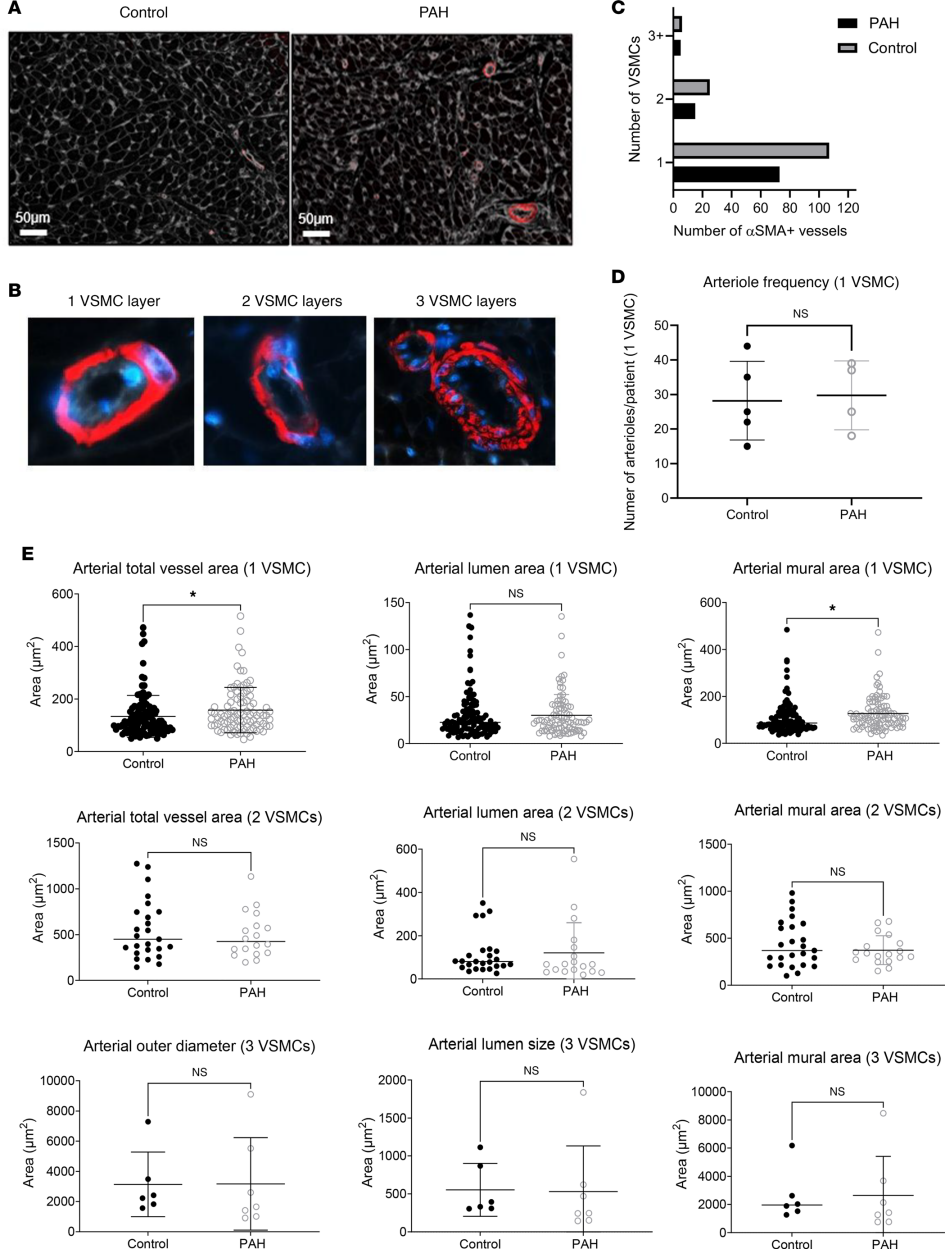
Histological analysis of vascular density and arteriole morphology. (**A**) Representative images of right ventricular tissue from IPAH and control patient groups stained with WGA (gray) and αSMA (red). (**B**) Cropped regions of interest from lower magnification images of arterioles classified by vascular smooth muscle cell (VSMC) number visualized via αSMA staining. (**C**) Quantification of the number of αSMA^+^ arterioles identified histologically and classified by VSMC number between control and IPAH right ventricular samples. (**D**) Quantification of the number of 1-VSMC arterioles identified per patient showed no significant difference between IPAH and controls (IPAH 28.2, control 29.75, unpaired 2-tailed *t* test *P* = 0.8363). (**E**) Quantification of the outer arterial area, inner lumen area, and mural area for all arterioles identified with 1, 2, or 3 VSMCs. One VSMC: outer arteriole area (control = 141.6 μm^2^, IPAH = 181.3 μm^2^, *P* = 0.01 unpaired *t* test; luminal arteriole area, control = 31.39 μm^2^, IPAH = 37.32 μm^2^, *P* = 0.1429 unpaired *t* test; mural area, control = 107.2 μm^2^, IPAH = 127.3 μm^2^, *P* = 0.0416 unpaired *t* test). Two VSMCs: outer arteriole area (control = 499.8 μm^2^, IPAH = 519.4 μm^2^, *P* = 0.8282 unpaired *t* test; luminal arteriole area, control = 109.32, IPAH = 143.7 μm^2^, *P* = 0.3433 unpaired *t* test; mural area, control = 435.5 μm^2^, IPAH = 372.2 μm^2^, *P* = 0.3446 unpaired *t* test). Three VSMCs: outer arteriole area (control = 3,132 μm^2^, IPAH = 3,198 μm^2^, *P* = 0.9694 unpaired *t* test; luminal arteriole area, control = 552.8 μm^2^, IPAH = 369.4 μm^2^, *P* = 0.3199 unpaired *t* test; mural area, control = 2,580 μm^2^, IPAH = 2,640 μm^2^, *P* = 0.1905 unpaired *t* test). *P < 0.05.

Cardiomyocyte cross-sectional area in patients with PAH and controls was 382.9 ± 117.6 μm^2^ and 230.7 ± 60.5 μm^2^, respectively (*P* = 0.0390, Figure 2, A and B). Cardiomyocyte cross-sectional area in patients with PAH showed greater variability (PAH SD 113.8 μm^2^ vs. control SD 52.63 μm^2^, *P* = 0.0192). Qualitative observation indicated that PAH hearts exhibited distinct regions of interstitial and perivascular fibrosis (example in Figure 2C). However, perhaps surprisingly, no significant difference in the overall fibrotic area was detected between PAH and controls (23.72% vs. 21.5%, *P* = 0.19) or in the overall variability (SD) of fibrosis (3.91% vs. 2.78%, *P* = 0.064, Figure 2D). Capillary density was significantly lower in patients with PAH compared with healthy individuals (139.3 ± 22.34 vs. 199 ± 30.45 number/ROI, *P* = 0.0138, Figure 3, A and B), with no difference in capillary density variance (45.86 ± 13.92 vs. 46.69 ± 14.95, *P* = 0.9414, Figure 3C), and capillary density was inversely correlated with cardiomyocyte cross-sectional area (*R*^2^ = 0.762, *P* = 0.0021) (Figure 3D). Arterioles were classified by the number of vascular smooth muscle cells using α-smooth muscle actin (αSMA) staining (Figure 4, A and B). No significant differences were found in the number or size of larger arterioles (2 or 3 vascular smooth muscle cells, all *P* values > 0.05) (Figure 4, C–E). However, pre-capillary arterioles with a single layer of vascular smooth muscle cells were hypertrophic in patients with PAH compared with controls (127.3 ± 9.8 μm^2^ vs. 107.2 ± 20.1 μm^2^, *P* = 0.041) (Figure 4E).

## Discussion

We undertook a multisystem investigation of coronary pathophysiology in IPAH. Based on in vivo and ex vivo studies involving patients with IPAH and controls, the key findings were as follows: first, coronary microvascular dysfunction was highly prevalent in the IPAH group: 73% of participants had an increased microvascular resistance in one or more coronary arteries, and half (55%) had a reduced coronary flow reserve. Coronary microvascular resistance and coronary flow reserve differed in patients with IPAH compared with control participants who underwent invasive endotyping and had a final diagnosis of noncardiac symptoms.

Second, the abnormalities in coronary microvascular resistance and vasodilatory capacity were similar in the left anterior descending or right coronary arteries. This finding contrasts with coronary physiology in other groups without IPAH, including individuals with angina and unobstructed coronary arteries, since coronary microvascular dysfunction tends to be more pronounced in the right coronary artery compared with the left coronary artery (11). This observation is consistent with prior studies using Doppler flow sensor-tipped wires (5, 12). The sample size of our cohort indicates further prospective studies are warranted to investigate the clinical significance of microvascular dysfunction in the left coronary artery in IPAH.

Third, invasive coronary function testing, including the infusion of intravenous adenosine, after clinically indicated right heart catheterization, was not associated with adverse events, and therefore, this diagnostic approach appears to be feasible and safe in this population when performed by experienced cardiologists.

Finally, histopathology demonstrated that PAH is characterized by reduced capillary density, which is inversely correlated with cardiomyocyte hypertrophy. The observed capillary rarefaction in the right ventricle may be linked to the increased microvascular resistance identified in the coronary physiology data. Nevertheless, further studies correlating histology and physiology in the same patients are required to confirm causality between structural and functional findings. Our assessment of capillary density in this distinct cohort was performed using 2D image analysis, and it is important to recognize that this approach in the absence of a stereological 3D reconstruction does not allow for microvascular morphology to be comprehensively assessed (13). Recently, a deep tissue imaging approach revealed that microvascular structure was affected in the pressure overloaded right ventricle as opposed to capillary rarefaction being observed. This highlights that morphological alteration is an important histopathological feature in addition to microvascular density.

Although fibrosis is an established phenotype within the right ventricle of patients with PAH and in preclinical models (14–21), we did not observe a significant overall global difference. However, examination of right ventricular myocardium stained with wheat germ agglutinin (WGA) revealed diffuse perivascular fibrosis within the PAH cohort compared with non-PAH controls. Furthermore, the degree of fibrotic deposition has previously been shown as a variable phenotype dependent on etiology of PAH (22), which may have been difficult to detect within our patient cohort (*n* = 4). In addition, small pre-capillary arterioles show hypertrophy in PAH, with an increased wall-to-lumen ratio and evidence of increased perivascular fibrosis compared with healthy controls, which corresponds to previously published data of structural adaptations of the right atrium in pulmonary hypertension (23). Our study provides potentially new pathophysiological insights, and the findings are potentially clinically relevant (Figure 1B).

Chest pain can be a common symptom in patients with IPAH; for example, in our group, 45% had a history of atypical angina (24, 25). Chest symptoms in patients with IPAH have been suggested to result from right ventricle ischemia and left coronary artery compression secondary to pulmonary artery dilatation (26). Right ventricle ischemia may result from a relative reduction in systemic arterial blood pressure and increased right ventricular pressure, which reduces the perfusion gradient (5, 27). Right ventricular hypertrophy and microvascular rarefaction are likely to contribute, as evidenced by the findings in the histological dataset (28, 29). Coronary microvascular dysfunction is increasingly recognized in the etiology of patients with angina. Most patients in our group had abnormal invasive indices of coronary microvascular function. All participants had unobstructed coronary arteries. Based on our findings, increased microvascular resistance and/or impaired vasodilatory capacity is a plausible cause of angina in patients with IPAH (6).

We observed that 73% of participants had elevated coronary microvascular resistance. The IMR has not previously been measured in patients with IPAH, to our knowledge. In comparison to our study, Ilsar et al. measured microvascular resistance and flow reserve in the pulmonary circulation in a primate model (9). They demonstrated progressive pulmonary obstruction in response to microsphere administration. We found 55% of patients had reduced coronary flow reserve. Similarly, Vogel-Claussen et al. found reduced myocardial perfusion reserve (assessed by stress perfusion cardiovascular MRI) in patients with IPAH (8).

We included a control group of comprehensively phenotyped patients in which invasive coronary artery function testing had excluded both obstructive epicardial disease and coronary microvascular dysfunction. Their final diagnosis was noncardiac chest pain. Compared with the control group, participants with IPAH had markedly higher IMR values, consistent with increased microvascular resistance. Although progressive pulmonary vascular resistance secondary to remodeling pre-capillary pulmonary arterioles is a hallmark of IPAH, our histology data demonstrates that similar structural changes also occur in the coronary microvasculature. The observed changes in coronary function may be a consequence of pulmonary hypertension, right ventricular remodeling, and capillary rarefaction. However, alternatively, the shared histopathological features could suggest a potential common underlying pathogenic mechanism. Previous research has suggested that overexpression of bromodomain protein 4 (BRD4) may act as a trigger for the calcification and remodeling processes in both vascular beds, possibly contributing to disease progression in IPAH (30). This highlights how shared pathophysiological mechanisms may influence both pulmonary and cardiac phenotypes.

In participants with IPAH, there was a directional change toward lower coronary flow reserve values (i.e., impaired vasodilatation), although this was not statistically significant. Abnormal coronary flow reserve has previously been observed in patients with systemic hypertension, even in the absence of coronary artery disease or LVH. This has been attributed to vascular remodeling and altered coronary hemodynamics (31). Finally, lower measured fractional flow reserve values were observed in the control group (although none below the clinical threshold of 0.80 used to define flow-limiting disease), consistent with diffuse atherosclerotic disease. Notably, the non-IPAH control group was older and had a higher proportion of arterial hypertension and dyslipidemia. Additionally, fractional flow reserve calculations assume minimal microvascular resistance. The higher fractional flow reserve observed in the IPAH group may also reflect the elevated microvascular resistance.

Thermodilution-derived indices of microcirculatory resistance are typically greater in the right coronary artery than in other territories. Our observation of no difference between right, left, and circumflex coronary arteries suggests a global increase in the IMR in IPAH and a remodeling mechanism that does not only affect right ventricular function, as might be expected in this patient group. Furthermore, this global change appears to affect both left and right ventricles, as supported by the reduction in both left and right ventricular ejection fraction. The observed global reduction in cardiac function may also be associated with the increase in filling pressure (left ventricular end-diastolic pressure) in the IPAH group. Interestingly, pulmonary capillary wedge pressure was not elevated in the IPAH group. The reason for this discordance between left ventricular end-diastolic pressure and pulmonary capillary wedge pressure is not clear. However, it may indicate abnormal left ventricle or left atrial compliance in this population (32). Clinically relevant differences in cardiac parameters and comparable abnormalities in both right and left coronary arteries raise further questions about whether coronary microvascular dysfunction in this population is a primary pathological process or a secondary consequence of pulmonary hypertension and ventricular remodeling.

Under normal conditions, extravascular compression by the left ventricular myocardium during systole results in left coronary artery flow reversal. Therefore, maximal left coronary artery flow occurs in early diastole. In contrast, because of the lower pressure generated by the usually thin-walled right ventricle, there is no early systolic flow reversal in the right coronary artery, and systolic blood flow contributes a relatively greater proportion of total coronary flow compared with vessels supplying the left ventricle (5). Phasic flow patterns in the right coronary artery become apparent in situations of coronary obstructive disease and in PAH (5, 12). Compared with the control group, patients with IPAH had approximately twice as large right ventricle mass and had reduced right ventricle ejection fraction. In the right coronary artery, we found a strong positive correlation between both IMR and right ventricle mass and a negative correlation between coronary flow reserve and right ventricle mass. Van Wolferen et al. similarly found an inverse relationship between right coronary artery flow and right ventricle mass in patients with IPAH (using a cardiovascular MRI–derived blood flow quantification method) (5). In contrast, we did not find a correlation between the invasive metrics of coronary artery function and mean pulmonary artery pressure or right ventricle ejection fraction. This suggests that alterations in coronary artery function may be an independent process of increased pulmonary vascular resistance.

In the IPAH group, 60% of patients had elevated myocardial T1 relaxation times, which was more prevalent than in the control group. In this setting, increased native myocardial T1 is most likely associated with myocardial fibrosis, a recognized finding in IPAH (33). Late gadolinium enhancement imaging may also be used to identify myocardial fibrosis on cardiovascular MRI but is less sensitive for diffuse fibrosis. Right ventricle insertion hyperenhancement is a typical finding in PAH and an independent marker of poor prognosis and is felt to reflect increased fibrosis at these points (34, 35).

Clinical implications include whether routine noninvasive assessments of coronary microvascular dysfunction, such as by quantifying myocardial blood flow using stress imaging, or selective invasive endotyping in patients with IPAH with angina might improve clinical risk stratification. Future studies should include a larger, multicenter PAH population, which would allow evaluation of early versus late stages of coronary microvascular dysfunction, the relationship between severity and established risk scores, or noninvasive prognostic markers. Such a study could reassess the external validity of our findings and associations between participants’ characteristics and prognosis. Furthermore, previous noninvasive measurements in PAH cohorts include oxygen-sensitive cardiac MRI and myocardial stress perfusion assessments, which have high detection sensitivity for CMD linked to right ventricle functional impairments in patients with PAH (36, 37). Correlative studies between the invasive measurements performed in our current investigation and noninvasive parameters would corroborate mechanistic insights into right ventricle remodeling, CMD, and reduced perfusion reserve. Linkage with in-depth microvascular morphology analysis would provide a comprehensive assessment of right ventricle myocardial and vascular pathological adaptations in IPAH.

### Limitations

Although the sample size is limited, the cases and controls were reasonably well matched, with the control group being older on average than the IPAH cases, mitigating age-dependent progression of IPAH as a confounding factor. The participants had undergone a detailed, multimodality clinical evaluation according to international guidelines (38). We used a bolus thermodilution method to assess coronary artery function, and continuous thermodilution and intracoronary Doppler flow assessment are alternative methods (38). We performed coronary vascular function testing in at least one main epicardial coronary artery, and where feasible in both the left anterior descending and right coronary arteries. Our findings support the safety of this approach. In control individuals, all coronary function tests were limited to the left anterior descending artery, which may introduce a potential bias when comparing coronary function between groups. However, in the multivessel analysis, no difference was observed between the function of the left and right coronary arteries (Supplemental Table 2). Future studies may prioritize 3-vessel assessments and potentially acetylcholine provocation testing. We adopted binary thresholds to define abnormal coronary artery function, in line with international guidelines. The 9-year survival rate of 45% of patients in this cohort aligns with contemporary IPAH registries, indicating that participants were representative of typical IPAH populations rather than a particularly high-risk group. Larger future studies could explore whether abnormal coronary microvascular function predicts prognosis. Although there is some debate about reference ranges, the coronary physiology observations in the IPAH group were different from the control group metrics (39). The group of individuals who underwent postmortem evaluation lacked in vivo coronary physiology data; however, our findings merit further investigations in cohorts with paired coronary microvascular function and microvascular structural analyses to establish mechanistic insight. Given the low prevalence of IPAH and the availability of right ventricular biopsy samples, the complementary histological analysis utilized available tissue from the broader group 1 PAH population to examine end-stage structural remodeling across PAH subtypes.

### Conclusions

In this study, abnormalities in invasive parameters of coronary artery function were frequently observed in patients with IPAH, with the degree of microvascular obstruction and vasodilatory impairment correlating with right ventricle mass. Histopathological analysis of right ventricle tissue revealed capillary rarefaction, along with hypertrophy and fibrosis of small pre-capillary arterioles. Right ventricle maladaptive remodeling in pulmonary hypertension appears to be associated with microvascular dysfunction, which in turn may contribute to myocardial ischemia and disease progression, warranting further investigation. Importantly, an adjunctive invasive coronary function testing protocol was feasible and safe in patients undergoing clinically indicated right heart catheterization. This approach provides a platform to determine whether therapies targeting PAH can enhance cardiac microvascular function.

## Methods

### Sex as a biological variable

Both male and female individuals were enrolled in this study. There is a greater incidence of IPAH within the female population, which is reflected in the percentages of participants included within this study (Table 1). Sex stratification analyses were not performed due to the statistical power requirement of our study group size.

### Study design

We undertook a systems medicine evaluation of coronary pathophysiology in IPAH in vivo and ex vivo and a cross-sectional, case-control design. The in vivo investigation involved right and left heart catheterization, invasive endotyping of coronary physiology, echocardiography, and cardiovascular MRI during 1 episode of inpatient care.

This in vivo multisystem evaluation was conducted in a national referral center for IPAH (Scottish Pulmonary Vascular Unit, Golden Jubilee National Hospital). Patients with known or suspected IPAH (i.e., group 1 PAH) (3, 4) who were undergoing de novo elective inpatient investigation were prospectively invited to participate.

In parallel, an IPAH case-control ex vivo study was undertaken in the Vrije Universiteit, Amsterdam University Medical Centre (UMC), and right ventricular myocardium and vascular histopathology was examined.

### Screening

Potentially eligible participants were provided with an information document, and written informed consent was required before participation in the clinical arm of this study. Inclusion criteria were confirmed or suspected IPAH undergoing clinically indicated right heart catheterization. Exclusion criteria were the following: (a) pulmonary hypertension due to other causes or contributed to by other causes; (b) angiographic evidence of obstructive epicardial coronary artery disease; (c) inability to give informed consent; (d) age under 18 years; (e) history of allergic reaction to intravenous contrast agents; (f) contraindications to the use of intravenous adenosine.

Non-IPAH group 1 PAH conditions were excluded based on either the clinical presentation or genetic testing. Eligibility criteria were further assessed considering findings from right heart catheterization. Participants were eligible to continue in the study if pulmonary arterial hypertension was confirmed (mean pulmonary artery pressure ≥ 20 mmHg, pulmonary arterial wedge pressure ≤ 15 mmHg, and pulmonary vascular resistance > 2.0 WU) (4). If right heart catheterization excluded IPAH, then patients were discontinued from the study. Participants with confirmed IPAH continued immediately to the invasive coronary artery function testing protocol. Baseline clinical data, including right heart catheterization findings, transthoracic echocardiography, 6-minute walk test, cardiopulmonary exercise testing (where possible), and cardiovascular MRI, were prospectively recorded.

### Control population

A contemporary control group (*n* = 15) of participants in the Coronary Microvascular Angina (CorMicA) trial (ClinicalTrials.gov NCT03193294) were included. These individuals underwent clinically indicated invasive functional coronary angiography for assessment of suspected cardiac chest pain.

The individuals included in this control group had a history of stable chest pain, unobstructed coronary arteries, normal invasive coronary function tests (i.e., IMR, coronary flow reserve, and acetylcholine provocation testing), and cardiovascular MRI (40). Based on the findings from this comprehensive, multimodality evaluation, the final diagnosis was noncardiac chest pain.

### Invasive endotyping

Participants were advised to abstain from caffeine for 24 hours before the procedure. We aimed to measure coronary vascular function in both the left and right coronary arteries. In general, the left anterior descending coronary artery was selected since this artery typically subtends the greatest proportion of myocardial mass. Coronary microvascular function was assessed in the right coronary artery wherever feasible.

Coronary microvascular function was assessed using a pressure- and temperature-sensitive guide wire (Abbott Vascular). Intracoronary nitrate (200 μg) is routinely given during coronary angiography to attenuate coronary tone. Therefore, phosphodiesterase type 5 inhibitor therapy was withheld on the day of the cardiac catheterization to avoid a hemodynamic interaction with intracoronary nitrate. The coronary guidewire was calibrated outside the body, equalized with aortic pressure at the coronary guide catheter ostium, and advanced to the distal third of the epicardial coronary artery undergoing interrogation. Resting thermodilution was performed using 3 sequential intracoronary 3 mL boluses of 0.9% saline at room temperature. Maximal hyperemia was induced during a 3-minute infusion of adenosine (140 μg/kg/min) via an antecubital or central vein. Thermodilution was then repeated under hyperemic conditions. Mean aortic and distal coronary pressures were recorded during rest and maximal hyperemia, and the mean resting and hyperemic transit times were derived.

The safety of invasive coronary function testing was prospectively assessed by recording any adverse events or serious adverse events.

### Coronary physiology analysis

Coronary physiology recordings were analyzed offline (Coroventis) by an experienced observer (one of the authors). The invasive measurements of epicardial and microvascular function were the following: (a) fractional flow reserve, which describes the functional significance of an epicardial stenosis — fractional flow reserve = distal coronary pressure / aortic pressure, at maximal hyperemia (41); (b) coronary flow reserve, which reflects the vasodilatory capacity of the epicardial and microvascular coronary compartments — coronary flow reserve = mean resting transit time / mean hyperemic transit time (42) and is defined in AU; (c) IMR, a measure of coronary microvascular resistance — IMR = distal coronary pressure × mean transit time, at maximal hyperemia (13), which is defined in AU.

The IMR was corrected using Yong’s formula (IMR_calc_) and adjusted for invasively measured central venous pressure (right atrial mean pressure, mmHg) (IMR_cor_) (43, 44). The angiograms were analyzed by trained observers using imaging postprocessing software (QAngio XA, Medis).

### Definitions of endotypes of abnormal coronary vascular function

A fractional flow reserve threshold of 0.80 or less defined an artery with flow-limiting epicardial coronary artery disease (45). Fractional flow reserve was corrected for central venous pressure (right atrial mean pressure, mmHg) where available (46). Microvascular dysfunction was defined as increased microvascular resistance (IMR_cor_ ≥ 25), and/or impaired coronary vasodilator reserve (coronary flow reserve < 2.0) (47, 48).

### Cardiovascular MRI

#### Acquisition.

Participants underwent multiparametric cardiovascular MRI at 1.5 Tesla (Siemens MAGNETOM Avanto) 72 hours before the right heart catheterization. The imaging protocol included steady-state free precession cine-imaging of left and right ventricular mass and function, tissue characterization (T1 mapping), and late gadolinium enhancement imaging 15 minutes after administration of gadolinium contrast media (Gadovist, 0.2 mmol/L; Bayer). All the scan acquisitions were spatially coregistered.

#### Analysis.

The cardiovascular MRI analyses were performed on QMass 7.6 (Medis). Postprocessing was performed as per international recommendations (49). Cardiovascular MRI analyses were performed by 2 of the authors, who were blinded to the invasive findings.

#### Transthoracic echocardiography.

Participants underwent clinically indicated transthoracic echocardiography to derive measurements of right ventricular structure and function: tricuspid regurgitation peak gradient (mmHg), right ventricular end-diastolic diameter (cm), and tricuspid annular plane systolic excursion (cm). The imaging and analysis were performed by sonographers accredited by the British Society of Echocardiography who were blinded to the invasive findings.

The primary outcome was the proportion of patients with abnormal coronary vascular function, defined as either an increased IMR (≥25) or reduced coronary flow reserve (<2.0).

The secondary outcomes and analyses were the following: (a) feasibility and safety of routine coronary artery function testing during standard clinically indicated left and right heart catheterization; (b) comparison of left anterior descending and right coronary artery microvascular function; (c) associations between baseline patient factors and abnormal coronary microvascular function; (d) associations between right heart catheterization, cardiovascular MRI data, and abnormal coronary microvascular function.

### Microvascular structural characterization

Tissue processing and image acquisition were performed in Amsterdam UMC, and image analysis was performed in Glasgow, with the researchers blinded to the group assignments.

### Immunohistofluorescence

Right ventricular tissue comprising endocardial, myocardial, and epicardial layers from the midmyocardial free wall of control deceased individuals (*n* = 5) and deceased or transplanted patients with PAH (*n* = 4) were fixed in 4% paraformaldehyde (PBS, pH 7.4) for 24 hours before being embedded in paraffin. The paraffin blocks were then sectioned into 5 μm slices and mounted onto slides coated with 3-aminopropyl-triethoxysilane. The sections were dewaxed through a series of washes: two 3-minute xylene washes, one 3-minute xylene-ethanol wash, and graded ethanol washes (100% twice, followed by 95%, 70%, and 50%), finishing with a rinse under cold running water. Antigen retrieval was carried out by steaming the sections in a citrate-based solution (pH 6) under high pressure for 30 minutes. After permeabilization and blocking with 10% horse serum, the sections were incubated for 1 hour at room temperature with Cy3-conjugated antibody against αSMA (1:200 dilution, Sigma-Aldrich). Counterstaining was performed using Hoechst 33342 (1:1,000 dilution, Santa Cruz Biotechnology) and Alexa-647-conjugated WGA (1:200 dilution, Thermo Fisher Scientific). The samples were cover-slipped and mounted with ProLong Gold Antifade Mountant (Thermo Fisher Scientific). Whole-slide image acquisition was performed on Vectra Polaris (Akoya) at 20× magnification, and multispectral fields were acquired at 40× magnification. Tissue autofluorescence was removed before quantifications with inForm Tissue Analysis software (Akoya) with the specific spectral library for the fluorescent antibodies used. Cardiomyocyte cross-sectional area, vessel density, and fibrosis (WGA positive) area were quantified using ImageJ (NIH), with the classification of the vasculature (based on vascular smooth muscle cell layers), averaging data from 10 randomly selected regions of interest (ROI) per patient. WGA staining has been previously reported to have strong positive correlation with myocardial fibrosis stains, such as Picrosirius red and fibronectin immunolabeling, and was utilized to quantify fibrosis as was already performed for cardiomyocyte cross-sectional area analysis (50, 51).

### Image analysis

Arterioles positively stained for αSMA-555 were screened, and then total, mural, and lumen areas were manually quantified using the ImageJ (NIH) analysis platform. Capillary density was quantified using ImageJ Analyze Particles plug-in. Ten ROI per patient underwent threshold transformation on WGA-positive images, which identified membrane staining. To isolate cardiomyocytes/vascular smooth muscle cells from endothelial staining, the particle size threshold was set at 2–50 μm^2^ with a circularity parameter of 0.5–1 arbitrary units to discount background/nonspecific staining from analysis. Overlay masks were generated following discrimination of capillary structures, which met set parameter limits.

### Statistics

Data are presented as mean (± SD) or *n* (%) where appropriate. Groups were compared using Fisher’s exact tests for categorical variables and paired samples using 2-tailed *t* tests or Wilcoxon tests for continuous variables. Parametric tests, (i.e., unpaired 2-tailed *t* tests) were employed upon analysis and confirmation of normality using Shapiro-Wilk tests. Simple linear regression analysis was performed to assess the relationship between the continuous variables and presented with 95% CI as appropriate. Statistical analyses were performed using SPSS version 26 and GraphPad Prism 10. All tests were 2-sided, and a *P* value less than 0.05 was considered statistically significant.

### Study approval

The West of Scotland Research Ethics Service approved the study (reference 15/WS/0134). The use of explanted and postmortem human right ventricle autopsy samples was authorized by the ethics committee of Amsterdam UMC and was conducted in accordance with the principles of the Declaration of Helsinki. The Medical Ethics Review Committee of Amsterdam UMC determined that the study did not fall under the Medical Research Involving Human Subjects Act (WMO), hence informed consent was not required for the histological arm of this study (approval number 2012288).

### Sample size calculation

Given that the study population was restricted to IPAH, which is a rare disease, we estimated that data in at least 10 individuals would provide reasonable estimates of the range of values and variance, providing indicative, representative measurements that might be observed in a larger population of patients with IPAH. Considering the primary endpoint of difference in microvascular resistance, we anticipated a large effect size (difference between the groups) of 1.25, and a 2-tailed test (α = 0.05) estimated that 12 patients in each group would give 80% power to detect a between-group difference in IMR.

### Data availability

Data are available in the Supporting Data Values file.

## Author contributions

CB was responsible for study concept and design. EB, MGF, DSC, and CB prepared the manuscript and supplementary documents. All authors reviewed and provided edits to the final manuscript. DSC, TJF, BH, DC, KPB, MJ, CC, and CB collected and processed data and conducted subsequent analyses pertaining to clinical investigation. EB and MGF performed statistical analysis of clinical datasets. ALV and FSDM acquired and processed patient histopathological samples. EB and MGF conducted histopathological analyses.

## Funding support

- British Heart Foundation (BHF) (PG/18/6134217), (FS/14/15/30661) (to DSC), RE/13/5/30177; PG/17/2532884 (to TJF), and RE/13/5/30177; RE/18/6134217; FS/14/15/30661; FS/17/2632744; PG/17/2532884 (to CB).
- Dutch Heart Foundation (03-002-2023-0031, 02-001-2022-0123 to FSDM).
- Dutch Cardiovascular Alliance (PHAEDRA-IMPACT CVON-2018-29, DOLPHIN-GENESIS CVON-2017-10 to FSDM).
- Netherlands Organization for Scientific Research (NWO-VICI 09150182310056 to FSDM).
- Golden Jubilee Research Foundation, study sponsor.

## Supplementary Material

Supplemental data

ICMJE disclosure forms

Supporting data values

## Figures and Tables

**Table 1:**
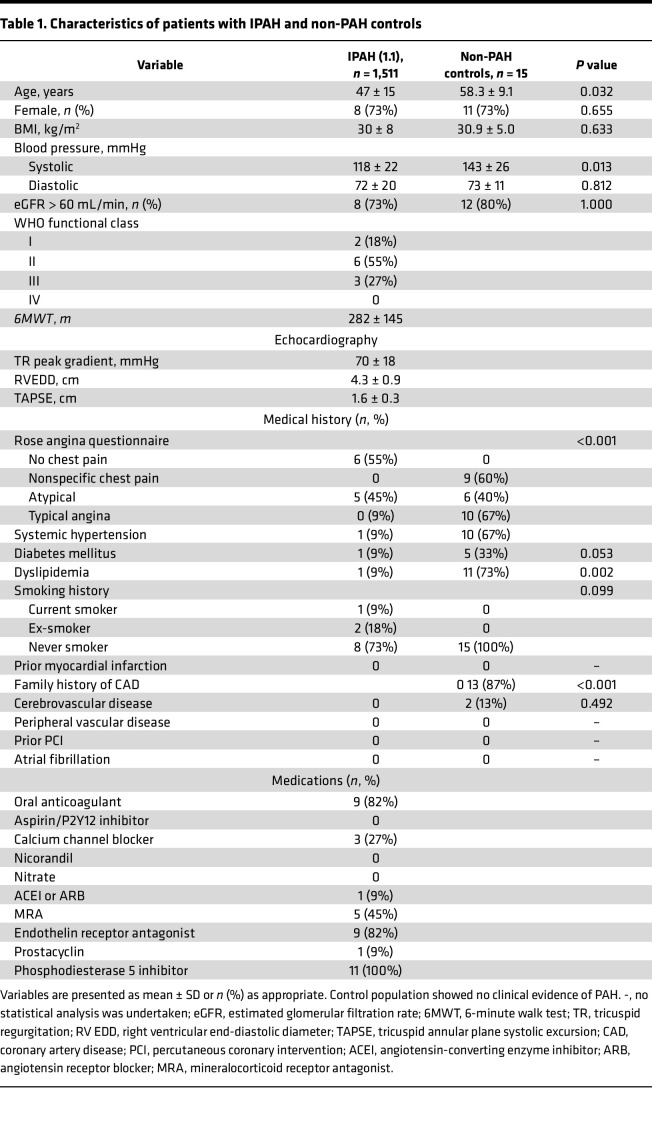
Characteristics of patients with IPAH and non-PAH controls

**Table 2:**
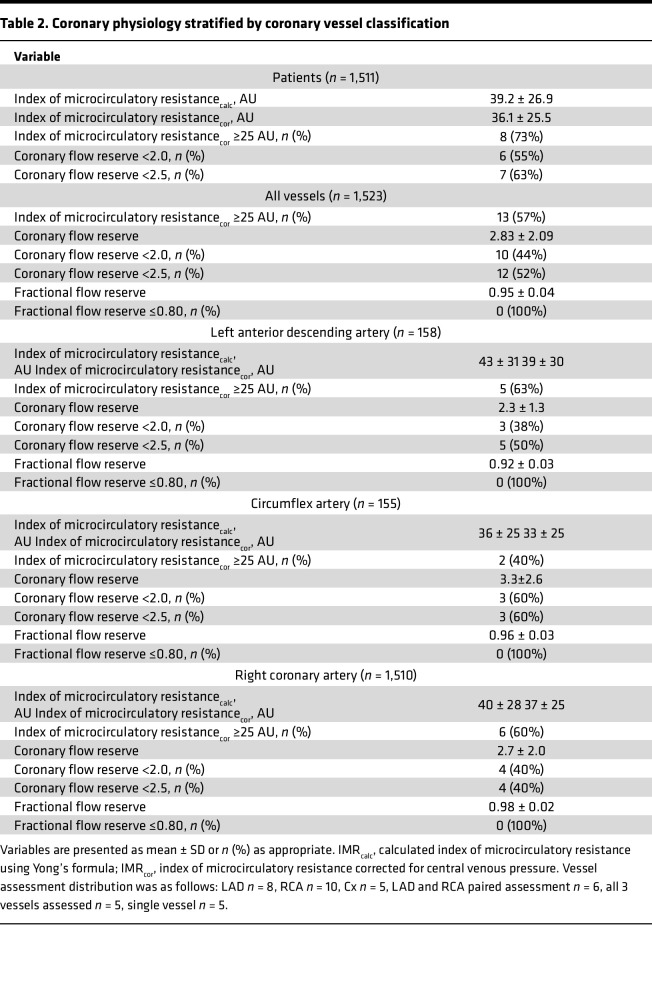
Coronary physiology stratified by coronary vessel classification

**Table 3:**
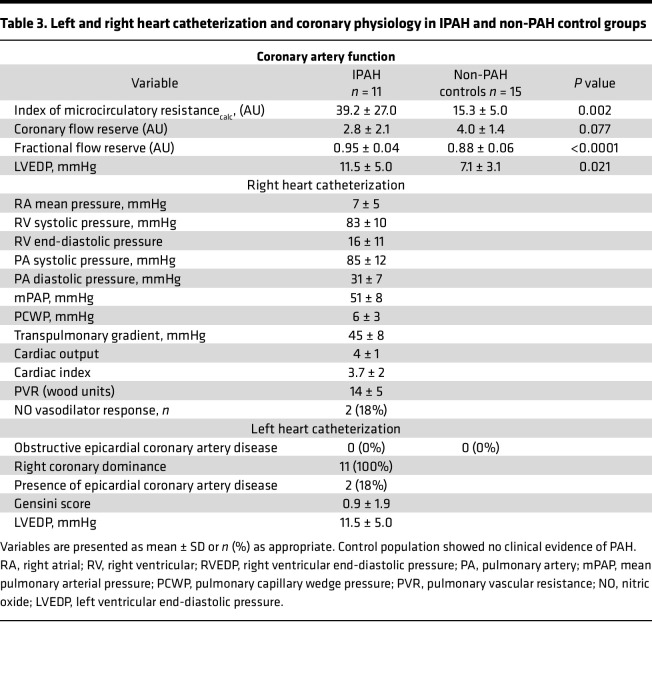
Left and right heart catheterization and coronary physiology in IPAH and non-PAH control groups

**Table 4:**
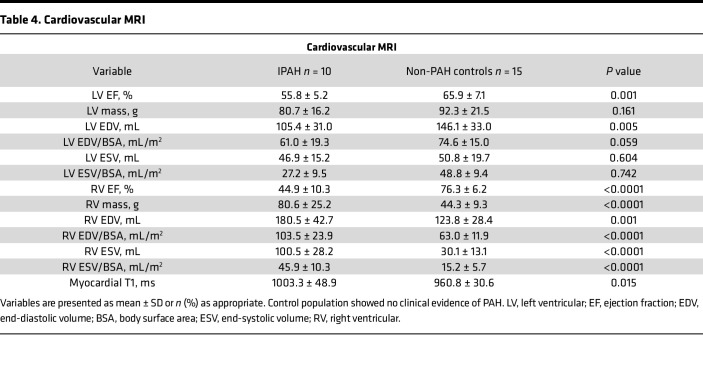
Cardiovascular MRI

## Version 1 01/22/2026

In-Press Preview

## Version 2 03/09/2026

Electronic publication

## References

[1] Badesch DB (2010). Pulmonary arterial hypertension: baseline characteristics from the REVEAL Registry. Chest.

[2] Ling Y (2012). Changing demographics, epidemiology, and survival of incident pulmonary arterial hypertension: results from the pulmonary hypertension registry of the United Kingdom and Ireland. Am J Respir Crit Care Med.

[3] Benza RL (2012). An evaluation of long-term survival from time of diagnosis in pulmonary arterial hypertension from the REVEAL Registry. Chest.

[4] Galiè N (2016). 2015 ESC/ERS Guidelines for the diagnosis and treatment of pulmonary hypertension: The Joint Task Force for the Diagnosis and Treatment of Pulmonary Hypertension of the European Society of Cardiology (ESC) and the European Respiratory Society (ERS): Endorsed by: Association for European Paediatric and Congenital Cardiology (AEPC), International Society for Heart and Lung Transplantation (ISHLT). Eur Heart J.

[5] van Wolferen SA (2007). Right coronary artery flow impairment in patients with pulmonary hypertension. Eur Heart J.

[6] Ford TJ (2018). Stable coronary syndromes: pathophysiology, diagnostic advances and therapeutic need. Heart.

[7] Wong YY (2011). Right ventricular failure in idiopathic pulmonary arterial hypertension is associated with inefficient myocardial oxygen utilization. Circ Heart Fail.

[8] Vogel-Claussen J (2011). Right and left ventricular myocardial perfusion reserves correlate with right ventricular function and pulmonary hemodynamics in patients with pulmonary arterial hypertension. Radiology.

[9] Ilsar R (2010). Measurement of pulmonary flow reserve and pulmonary index of microcirculatory resistance for detection of pulmonary microvascular obstruction. PLoS One.

[10] Ruiter G (2013). Right ventricular oxygen supply parameters are decreased in human and experimental pulmonary hypertension. J Heart Lung Transplant.

[11] Rehan R (2024). Multivessel coronary function testing increases diagnostic yield in patients with angina and nonobstructive coronary arteries. JACC Cardiovasc Interv.

[12] Seligman H (2022). Phasic flow patterns of right versus left coronary arteries in patients undergoing clinical physiological assessment. EuroIntervention.

[13] Ichimura K (2024). 3D imaging reveals complex microvascular remodeling in the right ventricle in pulmonary hypertension. Circ Res.

[14] Rain S (2013). Right ventricular diastolic impairment in patients with pulmonary arterial hypertension. Circulation.

[15] Omura J (2020). Identification of long noncoding RNA H19 as a new biomarker and therapeutic target in right ventricular failure in pulmonary arterial hypertension. Circulation.

[16] Crnkovic S (2019). Disconnect between fibrotic response and right ventricular dysfunction. Am J Respir Crit Care Med.

[17] Handoko ML (2009). Opposite effects of training in rats with stable and progressive pulmonary hypertension. Circulation.

[18] Andersen S (2019). Pressure overload induced right ventricular remodeling is not attenuated by the anti-fibrotic agent pirfenidone. Pulm Circ.

[19] van der Bruggen CE (2016). Bone morphogenetic protein receptor type 2 mutation in pulmonary arterial hypertension: a view on the right ventricle. Circulation.

[20] Overbeek MJ (2010). Characteristics of interstitial fibrosis and inflammatory cell infiltration in right ventricles of systemic sclerosis-associated pulmonary arterial hypertension. Int J Rheumatol.

[21] Gomez-Arroyo J (2014). Differences in right ventricular remodeling secondary to pressure overload in patients with pulmonary hypertension. Am J Respir Crit Care Med.

[22] Hsu S (2016). Right ventricular functional reserve in pulmonary arterial hypertension. Circulation.

[23] Wessels JN (2023). Right atrial adaptation to precapillary pulmonary hypertension: pressure-volume, cardiomyocyte, and histological analysis. J Am Coll Cardiol.

[24] Rich S (1987). Primary pulmonary hypertension. A national prospective study. Ann Intern Med.

[25] Torbicki A (2003). Detectable serum cardiac troponin T as a marker of poor prognosis among patients with chronic precapillary pulmonary hypertension. Circulation.

[26] Mesquita SMF (2004). Likelihood of left main coronary artery compression based on pulmonary trunk diameter in patients with pulmonary hypertension. Am J Med.

[27] Gómez A (2001). Right ventricular ischemia in patients with primary pulmonary hypertension. J Am Coll Cardiol.

[28] Bogaard HJ (2010). Adrenergic receptor blockade reverses right heart remodeling and dysfunction in pulmonary hypertensive rats. Am J Respir Crit Care Med.

[29] Fang Y-H (2012). Therapeutic inhibition of fatty acid oxidation in right ventricular hypertrophy: exploiting Randle’s cycle. J Mol Med (Berl).

[30] Meloche J (2017). Implication of inflammation and epigenetic readers in coronary artery remodeling in patients with pulmonary arterial hypertension. Arterioscler Thromb Vasc Biol.

[31] Camici PG, Crea F (2007). Coronary microvascular dysfunction. N Engl J Med.

[32] Hanna EB (2018). Mechanisms of discrepancy between pulmonary artery wedge pressure and left ventricular end-diastolic pressure in heart failure with preserved ejection fraction. JACC Heart Fail.

[33] Jayasekera G (2016). Right ventricular free wall myocardial tissue characterisation by systolic cardiac magnetic resonance T1 mapping in pulmonary hypertension. J Cardiovasc Magn Reson.

[34] Shehata ML (2011). Myocardial delayed enhancement in pulmonary hypertension: pulmonary hemodynamics, right ventricular function, and remodeling. AJR Am J Roentgenol.

[35] Freed BH (2012). Late gadolinium enhancement cardiovascular magnetic resonance predicts clinical worsening in patients with pulmonary hypertension. J Cardiovasc Magn Reson.

[36] Sree Raman K (2021). Right ventricular myocardial deoxygenation in patients with pulmonary artery hypertension. J Cardiovasc Magn Reson.

[37] Sree Raman K (2019). Feasibility of oxygen sensitive cardiac magnetic resonance of the right ventricle in pulmonary artery hypertension. Cardiovasc Diagn Ther.

[38] Knuuti J (2020). 2019 ESC Guidelines for the diagnosis and management of chronic coronary syndromes. Eur Heart J.

[39] Ong P (2018). International standardization of diagnostic criteria for microvascular angina. Int J Cardiol.

[40] Ford TJ (2018). Stratified medical therapy using invasive coronary function testing in angina: the CorMicA trial. J Am Coll Cardiol.

[41] Pijls NHJ (1996). Measurement of fractional flow reserve to assess the functional severity of coronary-artery stenoses. N Engl J Med.

[42] Gould KL (1974). Physiologic basis for assessing critical coronary stenosis. Instantaneous flow response and regional distribution during coronary hyperemia as measures of coronary flow reserve. Am J Cardiol.

[43] Fearon WF (2003). Novel index for invasively assessing the coronary microcirculation. Circulation.

[44] Yong AS (2013). Calculation of the index of microcirculatory resistance without coronary wedge pressure measurement in the presence of epicardial stenosis. JACC Cardiovasc Interv.

[45] Montalescot G (2013). 2013 ESC guidelines on the management of stable coronary artery disease. Eur Heart J.

[46] Pijls NHJ (2000). Coronary pressure measurement to assess the hemodynamic significance of serial stenoses within one coronary artery: validation in humans. Circulation.

[47] Lee JM (2016). Coronary flow reserve and microcirculatory resistance in patients with intermediate coronary stenosis. J Am Coll Cardiol.

[48] Lee B-K (2015). Invasive evaluation of patients with angina in the absence of obstructive coronary artery disease. Circulation.

[49] Schulz-Menger J (2020). Standardized image interpretation and post-processing in cardiovascular magnetic resonance - 2020 update: Society for Cardiovascular Magnetic Resonance (SCMR): Board of Trustees Task Force on Standardized Post-Processing. J Cardiovasc Magn Reson.

[50] Emde B (2014). Wheat germ agglutinin staining as a suitable method for detection and quantification of fibrosis in cardiac tissue after myocardial infarction. Eur J Histochem.

[51] Sedmera D (2024). Fibrosis and expression of extracellular matrix proteins in human interventricular septum in aortic valve stenosis and regurgitation. Histochem Cell Biol.

