# Supplementary material for: Coronary pathophysiology in idiopathic pulmonary arterial hypertension
Source: JCI Insight. 2026 Jan 22;11(5):e194613. doi: 10.1172/jci.insight.194613 (PMC13041685; doi:10.1172/jci.insight.194613)
Supplement: Supplemental data [file jciinsight-11-194613-s093.pdf]

Supplement

Full title

Coronary Pathophysiology in Idiopathic Pulmonary Arterial Hypertension: A Systems Medicine Study

Short title

Coronary Pathophysiology in IPAH

Supplementary Methods

CMR imaging

CMR imaging was performed on a Siemens AVANTO (Erlangen, Germany) 1.5 T MRI scanner with a 12-element phased array cardiac surface coil.[1] All patients underwent a standardised CMR protocol. All scan acquisitions were spatially co-registered. The imaging protocol consisted of: 1. Low-resolution scout images localizers; 2. Fast gradient echo ‘white blood’ images in the axial, coronal and sagittal planes. These fast, free-breathing images were analysed for extra-cardiac anatomy and pathology; 3. Steady-state free procession (SSFP) ‘cine’ imaging using a trueFISP sequence (multi-slice single-shot breath-hold true fast imaging). Cine imaging was performed in the 3 long-axis planes (HLA, horizontal long-axis; VLA, vertical long-axis; ‘LVOT’, left ventricular outflow tract/3-chamber view). A short axis cine ‘stack’ was then performed from the left ventricular base to apex using 7 mm slice thickness with a 3 mm inter-slice gap. The short axis stack was planned from the end-diastolic HLA and VLA cine images. Typical cine imaging parameters were: TE 1.2 ms, TR 3.3 ms, flip angle 70°, field of view 340 x 270 mm, matrix size 256 x 180; 4. Native T1 mapping was performed using a MOLLI investigational prototype sequence.[2] T1 maps were acquired in the HLA and 3 short-axis (base, mid and apical) planes. Native T1 images were performed prior to gadolinium contrast administration. Spatially co-registered images were then obtained approximately 15 mins following gadolinium contrast administration (at the end of the imaging protocol) to allow for myocardial ECV estimation. T1 imaging parameters were: bandwidth 1090 Hx/pixel, TE 1.1 ms, T1 of first experiment 100 ms, TI increment 80 ms, flip angle 35°, matrix size 192 x 124 pixels; spatial resolution 2.1 x 1.1 x 8.0 mm; slice thickness 8 mm; scan time 17 heartbeats.

CMR image analysis

The CMR analyses for LV volumes and function, and T1 were performed on QMass 7.6 (Medis, Leiden, Netherlands). Post-processing was performed as per international recommendations.[3]

All CMR analyses were performed by a European Association of Cardiovascular Imaging (EACVI) Level 3 accredited physician with >3 years CMR experience.

Left and right ventricular volumes were calculated by manually contouring the end-diastolic and end-systolic short axis cine frames. Approximately 11 short-axis cine slices allowed for complete ventricular coverage. The end-diastolic and end-systolic frames were chosen as the images with the qualitatively largest and smallest LV and RV dimensions, respectively. The most basal LV slice was determined by the most basal LV slice which still had a >50% circumference of myocardium around the LV blood pool. Papillary muscles were included in the LV blood pool. Following endocardial and epicardial contouring, an automated calculation by the QMass 7.6 software determined LV and RV volumes (absolute and indexed to body surface area), mass and ejection fraction (by a sum of discs method in which the LV volumes are determined from endocardial surface area from each slice are multiplied by the inter-slice distance).

Post-processing of the myocardial native T1 maps was performed as per international guidelines.[4] Prior to contouring, the raw T1 images were assessed for cardio-respiratory motion artefact and if significant artefact was present, then the segments were not included. The endocardial and epicardial borders and LV blood pool were manually contoured on the raw T1 pre- and post-gadolinium contrast maps (**Error! Reference source not found.**). A region-of interest was then manually drawn in the septum on the T1 maps. For the MOLLI investigational prototype sequence run on the 1.5T MRI scanner at our institution, the septal myocardial native T1 and ECV was found to be 940.5±23.6 ms.[5] Threshold values 2 x SD were used for defining abnormal T1 (987.7 ms).

Quantitative Coronary Angiography

QCA was performed by an experienced cardiologist in the Glasgow Angiography Core Laboratory, using proprietary automated edge-detection software (Medis QAngio XA 7.3, Leiden, Netherlands). The angiographic images were calibrated to the coronary guide catheter

size. The end-diastolic angiographic image demonstrating the best luminal contrast opacification was chosen for analysis.

Obstructive epicardial CAD was defined as any epicardial coronary stenosis with FFR ≤0.80, or if FFR measurement was not performed a percentage diameter stenosis of ≥70% in 1 angiographic view or ≥50% in 2 orthogonal views assessed by quantitative coronary angiography (QCA).[6] The Gensini score was calculated to quantify the epicardial atherosclerotic disease burden.

Supplementary results

Cardiovascular magnetic resonance imaging and coronary artery function

There were no correlations between left anterior descending index of microcirculatory $resistance_{cor}$ or coronary flow reserve and right ventricle mass (all p values >0.05). Right ventricle ejection fraction and mean pulmonary artery pressure did not correlate with the invasive parameters of coronary artery function (all p values >0.05). Myocardial native T1 relaxation (ms) and right ventricle mass were not correlated (r=0.67, p=0.15).

Supplementary Tables & Figures

Supplementary Table 1. Summary data of structural histopathological analysis.

Variable	IPAH	Control	p-value
**Patient characteristics**			
Age (years)	62.25 ± 7.45	42.4 ± 16.31	-
Gender (% F:M)	40/60	50/50	-
Number in group	4	5	-
**Clinical history**			
Comorbidities	Kidney failure (n=1)	-	-
	Obstructive sleep apnea (n=1)	-	-
	Hypertension (n=1)	-	-
	Valve disease (n=1)	-	-
	Diabetes (n=1)	-	-
	Osteoporosis (n=1)	-	-
	Atrial fibrillation (n=1)	-	-

	Breast cancer (n=1)	-	-
	**PAH etiology/cause of death**		
Diagnosis	1.2.1 Heritable PAH (HPAH) (n=1)	Brain trauma (injury) (n=1)	-
	1.1.1 Idiopathic PAH (n=2)	Intracerebral hemorrhage (n=2)	-
	1.4.1 PAH associated with CTD (n=1)	Traumatic injury (n=1)	-
		Bilateral pneumonia (n=1)	-
	**Clinical management**		
Medications	Bosentan (n=3)	-	-
	Sildenafil (n=4)	-	-
	Epoprostenol (n=1)	-	-
	Treprostinil (n=1)	-	-
	Diuretic (n=1)	-	-
	**Indices of cardiac and pulmonary function**		
NHYA	IV (n=4)	-	-
Cardiac Index	2 ± 0.424 (2/4)	-	-
mRAP (mmHg)	15 ± 8.49 (2/4)	-	-
PVR (dynes/sec/cm^{-5})	12.5 ± 4.278 (2/4)	-	-

mPAP (mmHg)	61± 4.242 (2/4)	-	-
NT-proBNP (pg/mL)	13,970 ± 5972.22 (2/4)	-	-
**Histological parameters**			
Heart weight (g)	709 ± 154.149	-	-
Cardiomyocyte CSA (μm^2)	382.9 ± 117.6	230.7 ± 60.47	**0.0390***
Capillary density/ROI	139.3 ± 22.34	199 ± 30.45	**0.0138***
Arteriole total area (1 VSMC) (μm^2)	157.4 ± 11.65	133.7 ± 23.77	**0.0426***
Arteriole lumen area (1 VSMC) (μm^2)	30.07 ± 3.351	30.61± 0.5401	0.8721
Arteriole mural area (1 VSMC) (μm^2)	127.3 ± 9.820	107.2 ± 20.13	**0.0416***
Arteriole total area (2 VSMC) (μm^2)	493.0 ± 90.95	549.7 ± 56.68	0.5366
Arteriole lumen area (2 VSMC) (μm^2)	120.8 ± 35.48	114.2 ± 6.585	0.8537
Arteriole mural area (2 VSMC) (μm^2)	372.2 ± 66.17	435.5 ± 63.27	0.3446
Arteriole total area (3 VSMC) (μm^2)	3170 ± 1492	3132 ± 37.50	0.9804
Arteriole lumen area (3 VSMC) (μm^2)	529.9 ± 280	552.8 ± 22.89	0.9363
Arteriole mural area (3 VSMC) (μm^2)	2640 ± 1326	2580 ± 60.39	0.9645
WGA + staining (% area)	23.72 2 ±.202	21.5 2. ± 504	0.1905

NYHA – New York Heart Association, WGA – Wheat Germ Agglutinin, VSMC – vascular smooth muscle cell, mPAP – mean pulmonary artery pressure, mRAP mean right atrial pressure, NT-proBNP - N-terminal prohormone of brain natriuretic peptide, CSA – cross sectional area, PVR – pulmonary vascular resistance.

Table 2. multi-vessel analysis

Variable	Left anterior descending artery	Right coronary artery	p-value
IMR_{cor}, U	39±32	39±28	0.30
CFR	2.5±1.3	3.8±3.2	0.69
FFR	0.92±0.02	0.97±0.3	0.47

Variables are presented as mean±SD. The multivessel analysis includes the n=6 patients with invasive coronary artery function testing in both the left anterior descending and right coronary arteries.

IMR_{cor} = index of microcirculatory resistance corrected for central venous pressure, CFR = coronary flow reserve, FFR = fractional flow reserve.

Figure 1. Correlation of right ventricular mass with coronary microvascular resistance (a) and coronary flow reserve (b).

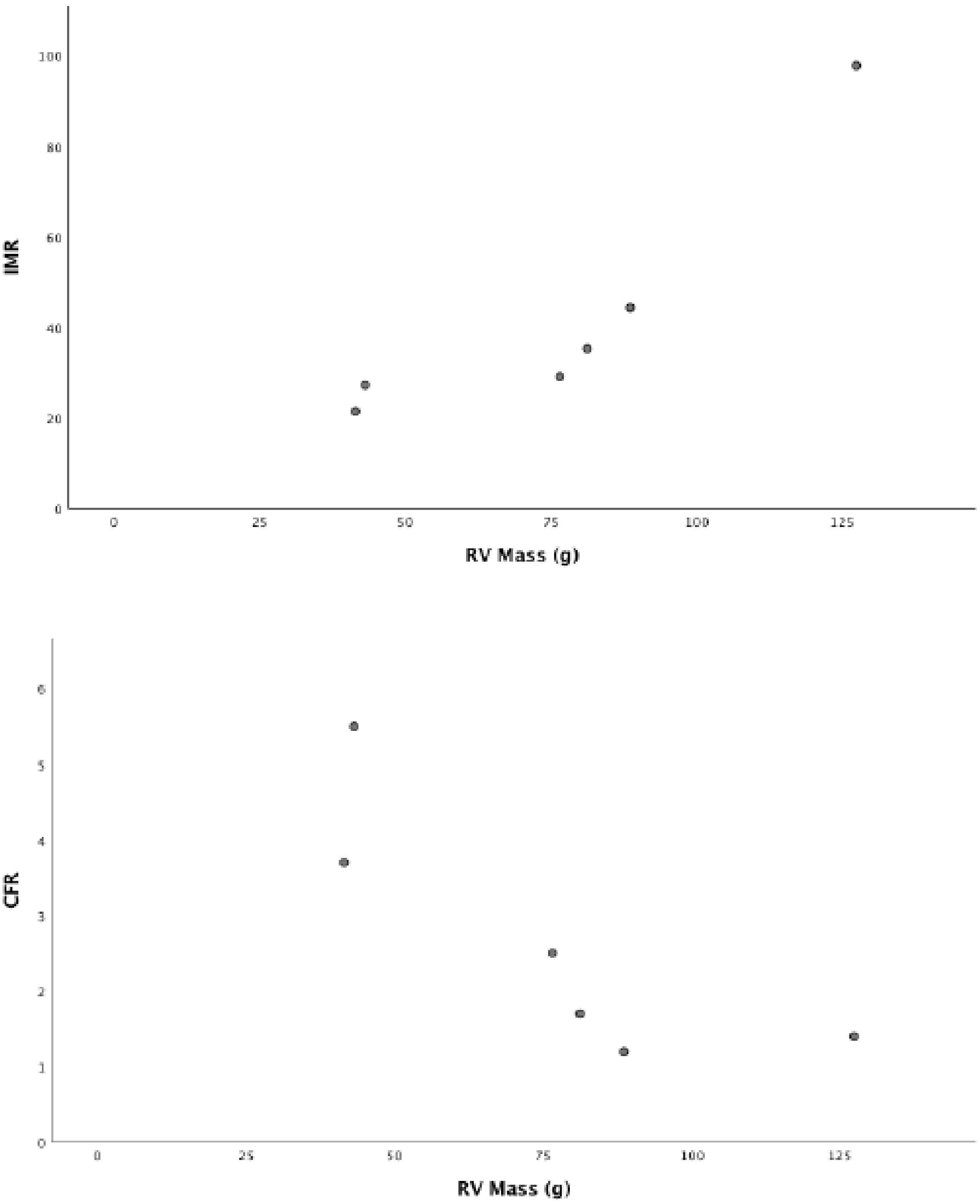

IMR = index of microcirculatory resistance corrected for central venous pressure, RV = right ventricular, CFR = coronary flow reserve.

Supplementary References

1. Kramer CM, Barkhausen J, Flamm SD, Kim RJ, Nagel E and Society for Cardiovascular Magnetic Resonance Board of Trustees Task Force on Standardized P. Standardized cardiovascular magnetic resonance (CMR) protocols 2013 update. *Journal of cardiovascular magnetic resonance : official journal of the Society for Cardiovascular Magnetic Resonance*. 2013;15:91.

2. Messroghli DR, Radjenovic A, Kozerke S, Higgins DM, Sivananthan MU and Ridgway JP. Modified Look-Locker inversion recovery (MOLLI) for high-resolution T1 mapping of the heart. *Magn Reson Med*. 2004;52:141-6.

3. Schulz-Menger J, Bluemke DA, Bremerich J, Flamm SD, Fogel MA, Friedrich MG, Kim RJ, von Knobelsdorff-Brenkenhoff F, Kramer CM, Pennell DJ, Plein S and Nagel E. Standardized image interpretation and post processing in cardiovascular magnetic resonance: Society for Cardiovascular Magnetic Resonance (SCMR) board of trustees task force on standardized post processing. *Journal of cardiovascular magnetic resonance : official journal of the Society for Cardiovascular Magnetic Resonance*. 2013;15:35.

4. Moon JC, Messroghli DR, Kellman P, Piechnik SK, Robson MD, Ugander M, Gatehouse PD, Arai AE, Friedrich MG, Neubauer S, Schulz-Menger J, Schelbert EB, Society for Cardiovascular Magnetic Resonance I and Cardiovascular Magnetic Resonance Working Group of the European Society of C. Myocardial T1 mapping and extracellular volume quantification: a Society for Cardiovascular Magnetic Resonance (SCMR) and CMR Working Group of the European Society of Cardiology consensus statement. *Journal of cardiovascular magnetic resonance : official journal of the Society for Cardiovascular Magnetic Resonance*. 2013;15:92.

5. Rauhalammi SM, Mangion K, Barrientos PH, Carrick DJ, Clerfond G, McClure J, McComb C, Radjenovic A and Berry C. Native myocardial longitudinal (T1) relaxation time: Regional, age, and sex associations in the healthy adult heart. *J Magn Reson Imaging*. 2016;44:541-8.

6. Tonino PA, De Bruyne B, Pijls NH, Siebert U, Ikeno F, van' t Veer M, Klauss V, Manoharan G, Engstrom T, Oldroyd KG, Ver Lee PN, MacCarthy PA, Fearon WF and Investigators FS. Fractional flow reserve versus angiography for guiding percutaneous coronary intervention. *The New England journal of medicine*. 2009;360:213-24.
